# A42 A HIGH SALT DIET INCREASES ELASTOLYTIC ACTIVITY AND WORSENS COLITIS IN GNOTOBIOTIC MICE

**DOI:** 10.1093/jcag/gwad061.042

**Published:** 2024-02-14

**Authors:** A Hann, M Bording Jorgensen, A Santiago Badenas, M Constante, K Jackson, P Bercik, H Galipeau, E Verdu

**Affiliations:** McMaster University, Hamilton, ON, Canada; McMaster University, Hamilton, ON, Canada; McMaster University, Hamilton, ON, Canada; McMaster University, Hamilton, ON, Canada; McMaster University, Hamilton, ON, Canada; McMaster University, Hamilton, ON, Canada; McMaster University, Hamilton, ON, Canada; McMaster University, Hamilton, ON, Canada

## Abstract

**Background:**

Recent evidence has linked ultra-processed foods, which are high in salt, to increased risk of inflammatory bowel disease (IBD). A high salt diet (HSD) was also shown to be colitogenic in specific pathogen-free mice, in part, through modulation of the microbiota. We recently described high microbial proteolytic activity (PA) in feces from IBD patients, that when transferred to germ-free mice, increased acute colitis severity. However, the drivers of high microbial PA are unknown.

**Aims:**

To determine whether HSD increases proteolytic activity, thereby exacerbating colitis.

**Methods:**

Adult germ-free (GF) C57BL/6 mice were colonized with feces from a patient with UC in flare (n=13). Mice were fed either a control diet (CD; 7004, Teklad) or a HSD (7004 supplemented with 4% NaCl) plus 1% NaCl in drinking water. Three weeks following colonization, chronic colitis was induced in half of the mice by three cycles (five days each; 2.0%, 1.5% and 1.5%, respectively) of DSS in drinking water with a five-day wash-out period between cycles. Fecal PA and predicted microbial protease profiles were measured before colitis induction. Weight loss and disease activity (e.g., occult blood in feces and stool consistency) was evaluated for the duration of colitis and microscopic colitis was evaluated at endpoint.

**Results:**

In mice colonized with UC microbiota, a HSD increased elastolytic activity (p=.03 versus CD) and induced a unique predicted protease and peptidase profile. After colitis induction, mice consuming a HSD lost more weight than mice consuming the CD (p=.0001) and had higher disease activity (p=.0004). Additionally, HSD induced more severe microscopic colitis scores compared with the CD (p=.0001).

**Conclusions:**

These results raise the hypothesis that a HSD drives microbial proteolytic activity of IBD microbiota. However, whether this shift is causally associated with the worsening of colitis remains unclear. Mechanistic insight underlying this HSD-microbial proteolytic function could lead to specific dietary modifications or anti-proteolytic therapies for IBD in patients with proteolytic-driven inflammation.

**Funding Agencies:**

CCC, CIHR

